# Deep brain stimulation for clozapine-resistant schizophrenia: a systematic review of target-specific outcomes and stereotactic technical considerations

**DOI:** 10.1007/s10143-026-04296-9

**Published:** 2026-05-02

**Authors:** Alivery Raihanada Armando, Achmad Fahmi, Heri Subianto, Agus Turchan, Wei Wang, Youheng Peng, Muhammad Fadhil Kamaruddin, Ramidha Syaharani, Ramadhani Rizki Zamzam, Nathania Maulina

**Affiliations:** 1 Faculty of Medicine, Airlangga University, Surabaya, Indonesia; 2 Department of Neurosurgery, Dr. Soetomo General Academic Hospital, Surabaya, Indonesia; 3 Department of Neurosurgery, West China Hospital, Sichuan University, Chengdu, China

**Keywords:** Deep brain stimulation, Treatment-resistant schizophrenia, Clozapine-resistant schizophrenia, Psychosurgery, Neurosurgical technique, Neuromodulation, Stereotactic surgery, neurosurgery

## Abstract

Treatment-resistant schizophrenia (TRS) affects approximately 20–30% of patients, and a substantial proportion develop clozapine-resistant schizophrenia (CRS). Deep brain stimulation (DBS) has emerged as a potential neurosurgical intervention targeting dysfunctional cortico–striato–limbic circuitry. However, technical heterogeneity and limited clinical data constrain interpretation of outcomes. This systematic review was conducted in accordance with PRISMA guidelines and prospectively registered in PROSPERO (CRD420251080715). Seven electronic databases were searched from inception through January 31, 2025. Clinical studies investigating DBS in TRS or CRS were included. Methodological quality was assessed using Joanna Briggs Institute (JBI) tools. Data extraction emphasized stereotactic targeting methods, hardware configurations, stimulation parameters, and clinical outcomes. The total number of study is 6. We excluded paper by Manssuer 2023. The total study population comprises 21 patients. Seven studies involving 21 patients met inclusion criteria. Targets included the nucleus accumbens (NAcc; n = 11), substantia nigra (SNr; n = 1), habenula (HB; n = 2), subgenual cingulate (SCG; n = 4), subgenual anterior cingulate cortex (sgACC; n = 3). Reported PANSS total score changes ranged widely (11%–85.7%), reflecting substantial inter-individual variability and methodological limitations. Surgical complications occurred in 3 of 21 patients (14.2%), including infection and hemorrhage. All cases utilized open-loop stimulation and conventional cylindrical leads. Current evidence suggests a preliminary therapeutic signal for DBS in highly selected CRS patients, particularly with NAcc targeting. However, conclusions remain limited by small sample sizes, technical heterogeneity, and absence of controlled trials. Future investigations should prioritize standardized stereotactic reporting, volumetric lead reconstruction, and long-term safety assessment within specialized neurosurgical research settings. PROSPERO CRD420251080715.

**Supplementary Information:**

The online version contains supplementary material available at 10.1007/s10143-026-04296-9.

## Introduction

Schizophrenia is a chronic psychiatric disorder affecting ~ 1% of the population, characterised by positive symptoms, such as hallucinations and delusions, and negative symptoms, such as lack of motivation and social withdrawal, alongside cognitive deficits that cause significant disability [35]. Around 20–30% of patients develop treatment-resistant schizophrenia (TRS), which is clinically defined as the persistence of significant symptoms despite at least two adequate trials of different antipsychotic medications of sufficient dose and duration [18]. TRS contributes to higher relapse rates, hospitalizations, and socioeconomic burden [20]. Although clozapine remains the gold standard, up to 60% fail to respond or tolerate it [38, 39]. For patients who fail to respond to or cannot tolerate clozapine, the more specific sub-specifier clozapine-resistant schizophrenia (CRS) is used. Approximately 40%–70% of TRS patients fall into this CRS category, which is associated with even greater symptom severity, higher relapse rates, and an increased socioeconomic burden [8, 25]. Augmentation strategies, such as electroconvulsive therapy (ECT) or repetitive transcranial magnetic stimulation (rTMS), provide inconsistent benefit, underscoring the need for novel therapies [31].

Deep brain stimulation (DBS), established in movement disorders [3], offers reversible modulation of pathological circuits and is increasingly explored in psychiatry. The rationale stems from circuit-based models of schizophrenia, which conceptualise the disorder as a disconnectivity syndrome involving a disintegration of mental processes [13]. This disintegration is hypothesized to be driven by an imbalance of neurotransmitters: dopaminergic hyperactivity in the mesolimbic pathway primarily underlies positive symptoms, whereas negative symptoms are associated with a “loss of information flow” or hypoactivity in the prefrontal cortex [15, 41]. Consequently, the surgical rationale for targeting the nucleus accumbens (NAcc) is to modulate the reward circuit and dampen hyper-dopaminergic signals driving psychosis, while targeting the medial prefrontal cortex (mPFC) or subgenual regions aims to restore network integration and alleviate negative symptoms [10, 16, 32].

The clinical success of DBS relies on precise anatomical targeting through sophisticated neuroimaging. Structural MRI at 3T (T1WI, T1IR, and STIR) and diffusion tensor imaging (DTI) enable the mapping of white matter tracts and microstructural architecture required to guide leads toward structures like the habenula and NAcc [2, 17, 22–24]. Target coordinate determination follows three primary approaches: statistical atlas-based coordinates, direct MRI visualization, or fusion of patient-specific imaging with stereotactic atlases [11, 27, 28]. While post-implantation verification and managing hardware-induced artifacts are critical for confirming electrode localization, the primary focus of the surgical intervention remains the stable modulation of the underlying dysfunctional circuitry [6, 33, 34, 37, 40].

The significant burden of clozapine-resistant schizophrenia (CRS) and the failure of standard augmentation strategies underscore the urgent need for therapeutic alternatives. Research indicates that additional antipsychotics are no more effective than placebo, and while options like mirtazapine or electroconvulsive therapy exist, clinical benefits remain inconsistent [44]. This lack of robust response necessitates circuit-based therapies like Deep Brain Stimulation (DBS) [31]. This systematic review evaluates the efficacy, safety, and surgical nuances across anatomical targets, including the NAcc and habenula, to establish a comprehensive framework for future neurosurgical trial designs targeting both TRS and CRS populations.

## Methods

### Study design and registration

This study is a systematic review conducted in accordance with the Preferred Reporting Items for Systematic Reviews and Meta-Analyses (PRISMA) guidelines (Page, McKenzie and Bossuyt, 2021). The review protocol was prospectively registered in PROSPERO (CRD420251080715).

### Information sources and search strategy

A comprehensive literature search was performed across seven electronic databases from inception to January 2025: PubMed (*n* = 804), Taylor & Francis Online (*n* = 654), ScienceDirect (*n* = 399), Scopus (*n* = 317), ProQuest (*n* = 172), Cochrane Library (*n* = 163), and ClinicalTrials.gov (*n* = 7). Additional records were identified through reference lists and manual searches (*n* = 5), yielding a total of 2,521 records.

To ensure transparency and reproducibility, specific Boolean search strings were tailored to each database. For instance, the PubMed search utilized: *schizophrenia[Title/Abstract] AND (“deep brain stimulation“[Title/Abstract] OR DBS[Title/Abstract] OR stimulation[Title/Abstract]) AND (outcomes OR efficacy OR safety)*. The Scopus search strategy employed: *(TITLE-ABS-KEY (schizophrenia) AND TITLE-ABS-KEY (“deep brain stimulation” OR DBS) AND ALL (outcomes OR efficacy OR safety))*. For the Cochrane Library, the search combined MeSH descriptors for [Deep Brain Stimulation] and [Schizophrenia] with keywords like “neuromodulation” to maximize sensitivity. No restrictions were placed on publication year.

### Eligibility criteria

Studies were eligible if they enrolled patients with a clinical diagnosis of schizophrenia or schizoaffective disorder (DSM or ICD) and were categorized into two distinct resistance profiles based on medication history. Treatment-Resistant Schizophrenia (TRS) was defined as the persistence of significant symptoms despite at least two adequate trials of different antipsychotic medications where clozapine had not yet been attempted or its failure was not explicitly documented. Conversely, Clozapine-Resistant Schizophrenia (CRS) was defined as the failure of at least two antipsychotics followed by an additional failed trial of clozapine at a therapeutic dose and duration. The intervention was limited to deep brain stimulation (DBS) delivered via implanted electrodes, including case reports, case series, and observational studies reporting original clinical data. Primary outcomes of interest included changes in psychiatric rating scales such as the PANSS or BPRS, while secondary outcomes focused on functional status and surgical safety parameters.

### Quality assessment

The methodological quality was appraised using the Joanna Briggs Institute (JBI) critical appraisal tools (Moola et al., 2020). Case reports, case series, and cross-sectional studies were evaluated using their respective 8-item or 10-item checklists. The JBI quality scores were used to qualitatively weight the evidence during data synthesis. Findings from studies that failed to meet key JBI criteria, such as clarity of inclusion criteria, measurement standardization, or the consecutive inclusion of participants, were interpreted with greater caution. These studies were categorized as providing “low-certainty” evidence, and their reported efficacy signals were moderated in the final discussion to avoid over-interpretation of results from methodologically limited data.

### Data synthesis

Data extraction focused on calculating the mean percentage improvement in PANSS scores across different anatomical targets to allow for standardized comparison. For studies reporting raw values, scores were converted to percentage reductions when mathematically feasible. Results were presented using descriptive tables summarizing patient characteristics, surgical technical parameters (coordinates, hardware, and targeting methods), and clinical outcomes. Given the significant heterogeneity in DBS targets (e.g., NAcc vs. Habenula), stimulation parameters, and the small sample size (*n* = 23), a quantitative meta-analysis was not feasible. Instead, results were synthesized narratively and organized by anatomical target to provide a target-specific assessment of therapeutic signals. Patterns related to follow-up duration and hardware-related complications were explicitly highlighted to inform future neurosurgical protocols.

## Results

### Study selection and characteristics

The systematic search identified seven clinical studies involving a total of 23 patients [1, 5, 7, 9, 26, 36, 42]. As summarized in Appendix: Table 1, the population was predominantly middle-aged with chronic illness durations ranging from 4 to 32 years. All demographic details, including gender and prior clozapine trials, are consolidated in Appendix: Table 1 to prioritize technical reporting.

The total study population comprises 23 patients (*n* = 23), categorized into two distinct categories: Clozapine-Resistant Schizophrenia (CRS) and Treatment-Resistant Schizophrenia (TRS). The CRS group (*n* = 21 patients) consists of chronic, middle-aged individuals (21–53 years) with illness durations reaching 32 years and documented failure of clozapine trials. Conversely, the TRS cohort (*n* = 2 patients) represents a younger demographic (16–21 years) without explicit clozapine resistance.

### Methodological quality assessment

Methodological quality was assessed via JBI critical appraisal tools as detailed in Appendix: Table 2. None of the studies met all high-quality criteria. Case reports generally provided strong clinical histories, but case series [1, 5] exhibited significant limitations in measurement standardization and statistical appropriateness. These methodological gaps necessitate a cautious interpretation of the reported efficacy signals.

### General neurosurgical technical parameters

Neurosurgical technical parameters for the 23 patients are summarized in Appendix: Tables 1 and 4. All studies utilized high-resolution 3.0T MRI for preoperative planning [5, 42]. Targeting strategies primarily involved MRI-direct visual targeting, though recent cohorts incorporated advanced tractography-activation modeling (TAM) to refine lead placement [1]. Quadripolar electrodes were used in 100% of cases, primarily Medtronic 3389 and 3387 leads. Notably, all included studies employed open-loop neuromodulation and conventional (non-directional) leads.

### Outcomes by anatomical target

1. Nucleus Accumbens (NAcc)

The NAcc was the most frequently evaluated region (*n* = 11). Mean total PANSS reductions ranged from 13.2% to 48.5% among responders across three studies [5, 36]. In the largest NAcc cohort [61], five of six patients (83.3%) achieved clinically significant improvement, defined as a ≥ 25 reduction in total PANSS score, with individual improvements of 48.5%, 30.4%, 46.2%, 26.8%, and 44.1% reduction. Conversely, one patient in this cohort showed a negligible 1.2% increase in symptoms. Bioque et al. [6] reported consistent total score reductions of 28.6%, 13.2%, and 14.9% across three patients. Positive symptom sub-scores demonstrated even more robust signals, with reductions reaching 61.5% and 66.7% in individual cases [9, 36].

2. Habenula (HB)

Habenula targeting (*n* = 2) produced the most contradictory therapeutic signals. One patient achieved a 31.7% reduction in total PANSS score, whereas the second patient experienced a 9.5% increase in symptoms [42]. This highlights significant variability in therapeutic response and the potential for symptom exacerbation depending on specific circuit modulation.

3. Substantia Nigra pars reticulata (SNr)

In the single patient (*n* = 1) targeting the SNr, a 52.4% reduction in the Brief Psychiatric Rating Scale (BPRS) total score was maintained at 12 months. This clinical response was further characterized by a complete resolution of hallucinations and delusions [7].

4. Subgenual Cingulate Gyrus (SCG)

SCG stimulation (*n* = 8) yielded more conservative results compared to NAcc modulation. Reductions in total PANSS scores ranged from 11.3% to 27.4% across seven patients, while one patient demonstrated a 6.8% increase in symptoms [1]. Most patients clustered within a 16% to 23% reduction range, indicating a modest but consistent therapeutic signal for this target.

## Discussion

The results of this systematic review identify a preliminary therapeutic signal for DBS in TRS and CRS, particularly within the NAcc and SNr targets. However, these observations should be interpreted with cautious interpretation. The JBI methodological appraisal reveals that the available evidence is characterized by low-level study designs, primarily case reports and small series, which are highly susceptible to publication bias and regression to the mean [1, 5]. Strong claims of efficacy are currently limited by these methodological failures, and the observed response rates likely represent hypothesis-generating data rather than definitive proof of clinical effectiveness.

### Surgical considerations and stereotactic technique

Schizophrenia is increasingly conceptualized as a systemic “circuit disorder” rather than a localized pathology, where clinical symptoms emerge from dysregulated neural pathways amenable to focal neuromodulation [30]. Consequently, the rationale for neuroanatomical target selection in clozapine-resistant schizophrenia (CRS) is heavily dictated by matching specific clinical phenotypes to their underlying neurobiological dysfunctions. The Nucleus Accumbens (NAcc) and ventral striatum are primarily targeted to address the hypoactivation of reward circuitry that correlates with negative symptoms, apathy, and anhedonia [14, 30]. Mechanistically, NAcc modulation has been shown in preclinical models to reverse aberrant hippocampal hyperactivity, a key driver of subcortical dopamine neuron dysregulation [12, 30].

In contrast, the Associative Striatum (AST) is theoretically better suited for patients with prominent positive symptoms, such as refractory hallucinations and delusions. PET imaging confirms that the hallmark presynaptic dopamine elevation in schizophrenia is concentrated in the AST rather than the ventral striatum, suggesting that targeting this region provides a more biologically precise intervention for psychosis [29, 30]. Furthermore, functional imaging findings—such as hippocampal hypermetabolism observed on PET or fMRI—can serve as a critical biomarker for selecting the ventral hippocampus (vHipp) or its regulatory nodes. This approach aims to correct the physiological “pacemaker” that drives downstream dopamine hyperactivity in the midbrain-limbic system [12]. Additional targets focus on specific symptom dimensions, such as the Subgenual Anterior Cingulate Cortex (sgACC) for correcting Default Mode Network (DMN) abnormalities linked to affective dysregulation [14], and the Habenula as a strategic node to modulate aberrant reward-prediction and punishment signalling [30].

The clinical success of these interventions is heavily predicated on the precision of lead placement and the optimization of the volume of tissue activated (VTA). A primary technical challenge is the historical heterogeneity in target definition. Traditionally, indirect targeting using statistical atlas-based coordinates referenced to the AC-PC line was the standard approach [11, 28]. However, because the anatomical boundaries of regions like the NAcc exhibit significant individual variability, recent surgical protocols have pivoted toward direct MRI-visual targeting to achieve superior delineation [5, 42]. The integration of tractography-activation models (TAM) represents a pivotal advancement, allowing surgeons to identify specific white matter “bottlenecks,” such as the medial forebrain bundle, that require modulation to ensure therapeutic efficacy [1, 22].

Maintaining stereotactic accuracy is further complicated by intraoperative “brain shift”, the physical displacement of parenchyma following dural opening due to CSF loss and pneumocephalus. As discussed by Ivan et al., (2014), even a 1–2 mm shift can move the target away from the planned trajectory, effectively displacing the lead’s center of gravity. This mechanical error likely underlies the “inaccurate lead placement” reported by Manssuer et al., [26] and provides a technical explanation for the contradictory clinical signals seen in habenula targeting [42]. Therefore, intraoperative verification via CT/MRI fusion or 3D-fluoroscopy is strongly recommended to ensure the “planned coordinates” derived from preoperative imaging align with the “final electrode position” [33, 34, 37].

Lead design and hardware selection further dictate the safety profile. All reviewed studies (*n* = 23) exclusively utilized conventional cylindrical leads (e.g., Medtronic 3387/3389), which deliver an axisymmetric, 360-degree electric field [5, 9]. A significant limitation of these leads is the lack of control over the VTA, which is directly related to clinical outcomes. Unintended current spread likely accounts for reported adverse events, such as the transient akathisia observed in NAcc stimulation [9] and autonomic fluctuations during habenula testing [42]. By contrast, directional electrodes with segmented contacts allow for “steering” the stimulation field in the horizontal plane [45]. While directional leads demonstrate higher performance robustness 1–2 mm outside the target [19], their smaller contact surface area produces a higher current density. Consequently, surgeons should reduce maximum intensity thresholds and revise programming algorithms to avoid potential tissue damage [45].

The heterogeneity of schizophrenia symptoms suggests that a personalized approach to targeting is essential for therapeutic success. The nucleus accumbens (NAcc) and ventral striatum are particularly relevant for patients characterized by negative symptoms and reward-processing deficits, as these regions act as central hubs for reward anticipation [14, 30]. Conversely, the associative striatum (AST) is better suited for patients with dominant positive symptoms, such as persistent hallucinations, given that presynaptic dopamine elevation is most concentrated in this region [30]. For those with affective dysregulation or comorbid depressive features, the subgenual anterior cingulate cortex (sgACC) remains a speculative but promising node within the Default Mode Network [14]. Furthermore, targeting the ventral hippocampus (vHipp) is rationalized for patients showing hippocampal hypermetabolism, aiming to correct the glutamate/GABA imbalance that drives downstream dopamine hyperactivity [12].

For reproducibility in future studies, systematic reporting of stereotactic parameters is essential. Investigators should document frame-based versus frameless techniques, AC–PC definition methodology, radial targeting error, final lead deviation from planned coordinates, and volumetric reconstruction of the volume of tissue activated (VTA). Without standardized reporting of these metrics, cross-study comparison remains limited and target-specific conclusions remain provisional.

### Technological evolution: open-loop to adaptive systems

A significant technological constraint is that 100% of current clinical evidence relies on open-loop stimulation, where the dose is static and fails to respond to the patient’s fluctuating psychotic state [1, 9]. While these systems provide continuous modulation, successful psychiatric DBS depends on integrating both anatomy and function at the circuit level [4]. The “missing link” in current practice is exemplified by Manssuer et al. [26], who recorded Local Field Potentials (LFPs) in the habenula but did not utilize these signals for real-time control. The next surgical evolution should be adaptive DBS (aDBS), using biomarkers like high-gamma activity to trigger stimulation only when clinically required [26].

Functional imaging (PET/fMRI) provides a framework for biomarker-driven target selection, where hippocampal hypermetabolism justifies modulating the temporal-striatal “pacemaker” [12] and DMN instability guides sgACC targeting for affective dimensions [14]. Such personalized approaches, including the identification of emotion-related prefrontal-insular networks [43], may help prevent adverse events such as the significant weight gain observed in SNr cases [7]. However, these models remain theoretical extrapolations; despite the recognized widespread dysconnectivity characteristic of schizophrenia [10], only a minority of the reviewed cohort (*n* = 21) underwent metabolic or connectomic analysis [1, 36]. Consequently, while early PET metabolic shifts are proposed for real-time stimulation adjustments [16, 32], physical lead localization remains the clinical gold standard, as the predictive value of metabolic biomarkers currently lacks standardized validation across studies [6, 34].

### Ethical safeguards and patient vulnerability

The ethical justification for utilizing deep brain stimulation (DBS) in clozapine-resistant schizophrenia (CRS) resides in the extreme clinical severity of the disorder and the exhaustion of all standard therapeutic alternatives. Clinicians must acknowledge that the current evidence base remains strictly at Class IV, consisting only of case reports and small case series, which necessitates a rigorous risk-benefit calibration during the informed consent process.

This framework requires a transparent contrast between the 14.2% cumulative risk of serious surgical complications, including intracranial hemorrhage and infection, and the profound long-term morbidity of untreated CRS. For this ultra-refractory population, the near-certainty of persistent functional decline, high relapse rates, and the debilitating metabolic side effects of high-dose antipsychotic polypharmacy often represent a greater threat to life expectancy than the statistically estimable risks of neurosurgery.

Given that the evidence base is currently limited to Class IV data, the justification for intervention rests on the exhaustion of all pharmacological gold standards. Consequently, while DBS represents a vital salvage therapy for the most refractory patients, its clinical application must be strictly limited to rigorous research protocols and mandated only within expert, multidisciplinary academic centers.

## Limitations

This systematic review is constrained by several methodological and interpretative limitations that warrant cautious interpretation. First, the evidence base remains extremely limited, consisting of only 23 patients across heterogeneous study designs, primarily single case reports and small case series. The absence of randomized controlled trials (RCTs) and the scarcity of prospective longitudinal data preclude causal inferences about efficacy.

Second, as noted in the JBI quality assessment, none of the included studies achieved high-quality scores across all methodological domains, suggesting a high risk of publication bias and regression to the mean.

Third, there was substantial variability in neurosurgical parameters, including stereotactic coordinates, lead models, and stimulation parameters [9, 26, 42].

This technical heterogeneity reduces the ability to identify standardized target-specific programming profiles. Fourth, outcome measures were inconsistently reported; while most studies utilized PANSS or BPRS scores, reporting outcomes as raw scores rather than percentage improvements complicates cross-study comparisons.

Fifth, follow-up durations were highly variable (6 to 36 months), potentially underestimating long-term hardware-related complications or delayed therapeutic signals.

Sixth, surgical safety data, including the 14.2% complication rate involving infection and hemorrhage, highlights the significant risks associated with this experimental intervention in a vulnerable psychiatric population.

Lastly, publication bias remains a significant concern; striking clinical improvements are more likely to be reported than non-responses, potentially inflating the perceived efficacy of NAcc and habenula targeting. These limitations underscore the early developmental stage of DBS for schizophrenia and emphasize that this procedure should remain confined to highly specialized research settings until further evidence is established.

### Future directions: toward mechanism-guided and adaptive neuromodulation

The next phase of DBS development for TRS and CRS will require a shift from purely anatomical targeting toward physiologically informed, dynamically responsive neurosurgical strategies. Several technological and conceptual advances are poised to reshape how stimulation is delivered and monitored in this population.

1. Hardware Evolution: From Omnidirectional to Segmented Steering

Current evidence is limited by the use of conventional cylindrical leads (e.g., Medtronic 3387/3389) that deliver an axisymmetric, 360-degree electric field. While these have shown early promise, the complex microanatomy of the NAcc and habenula leaves a narrow therapeutic window. The future implementation of directional leads, utilizing radially segmented contacts, will allow surgeons to shape the electrical field and selectively influence specific fiber bundles while avoiding off-target structures such as the internal capsule. This “current steering” is critical for avoiding the stimulation-induced side effects (e.g., akathisia) identified in this review [9]. However, this transition requires refined surgical programming, as segmented contacts produce higher current densities, necessitating revised safety thresholds to prevent local tissue damage [19, 45].

2. Adaptive Neuromodulation: Moving Beyond Open-Loop Constraints

The most critical technological frontier is the transition from open-loop to adaptive (closed-loop) DBS (aDBS). Currently, 100% of reviewed clinical evidence relies on open-loop stimulation, which delivers a static “dose” that cannot adapt to the fluctuating symptomatic states of schizophrenia. aDBS represents a natural next step, where stimulation is automatically adjusted in real time based on detected neural signatures. The “missing link” for this evolution is the identification of reliable electrophysiological biomarkers. Preliminary work by Manssuer et al., [26] has already identified high-gamma activity in the human habenula that tracks aversive states. Integrating such local field potential (LFP) sensing into implantable pulse generators would allow for “on-demand” modulation, potentially preventing the overstimulation of affective circuits and reducing the metabolic side effects seen in SNr and NAcc targeting [1, 7].

3. Multimodal Surgical Planning and Intraoperative Precision

Ultimately, the future of DBS for schizophrenia lies in the integration of structural, functional, and electrophysiological maps.

- Tractography-Activation Models (TAM): To identify critical white matter “bottlenecks” [1].
- Intraoperative LFP Monitoring: To capture real-time circuit states and confirm the physiological target [26].
- Robotic-Assisted Targeting: To mitigate the 1–2 mm errors caused by intraoperative brain shift and pneumocephalus (Ivan et al., 2014; [34]). This multimodal framework will allow clinicians to move beyond a “one-target-fits-all” paradigm toward mechanistic patient stratification, selecting the NAcc, habenula, SCG, or SNr based on each patient’s unique circuit imbalance.

4. Preferred Stimulation Site for Standardized Research

Given the substantial heterogeneity across targets and study designs, future multicenter investigations would benefit from initial standardization around a limited number of anatomically and physiologically justified nodes. Based on currently available data, the nucleus accumbens (NAcc) represents a rational candidate for prioritization in early standardized trials.

The NAcc occupies a central position within limbic–striatal circuitry, integrating dopaminergic, motivational, and affective networks implicated in schizophrenia pathophysiology. Across the reviewed cohort, NAcc stimulation demonstrated the most consistent exploratory signal of clinical benefit relative to other targets. While the associative striatum (AST) may offer pathophysiological specificity for positive symptoms, prioritizing the NAcc in early multicenter protocols may reduce technical variability and facilitate reproducible target-specific programming strategies.

This recommendation should not be interpreted as definitive target superiority but rather as a pragmatic step toward methodological consolidation in a field currently characterized by anatomical dispersion.

## Conclusion

This systematic review synthesizes available clinical data on deep brain stimulation (DBS) for treatment-resistant and clozapine-resistant schizophrenia, with particular emphasis on stereotactic technique, hardware configuration, and target-specific variability. Across 23 reported patients, exploratory signals of clinical improvement were observed, most consistently with nucleus accumbens (NAcc) targeting. However, the absence of controlled trials, limited cohort size, and methodological heterogeneity preclude definitive efficacy conclusions.

Technical variability—including target definition, lead selection, and stimulation parameters—likely contributes substantially to outcome dispersion. All published cases relied on open-loop stimulation with conventional cylindrical leads, underscoring the need for improved current steering, volumetric modeling, and adaptive programming strategies.

At present, DBS for schizophrenia should remain confined to specialized neurosurgical research settings. Future progress will depend less on expanding target diversity and more on improving stereotactic precision, reproducible lead reconstruction, standardized reporting metrics, and integration of multimodal imaging with electrophysiological validation.

## Supplementary Information

Below is the link to the electronic supplementary material.

Supplementary Material 1 (DOCX 907 KB)

## Data Availability

The data that support the findings of this study are available from the corresponding author upon reasonable request.

## Declarations

### Statements

This literature review did not require ethics committee approval as it involved only the analysis and synthesis of existing published literature.
