# Supplementary material for: Deep brain stimulation for clozapine-resistant schizophrenia: a systematic review of target-specific outcomes and stereotactic technical considerations
Source: Neurosurg Rev. 2026 May 2;49(1):389. doi: 10.1007/s10143-026-04296-9 (PMC13135008; doi:10.1007/s10143-026-04296-9)
Supplement: Supplementary file 1 — Supplementary Material 1 (DOCX 907 KB) [file 10143_2026_4296_MOESM1_ESM.docx]

Table. 1 Summary of included studies in the systematic review


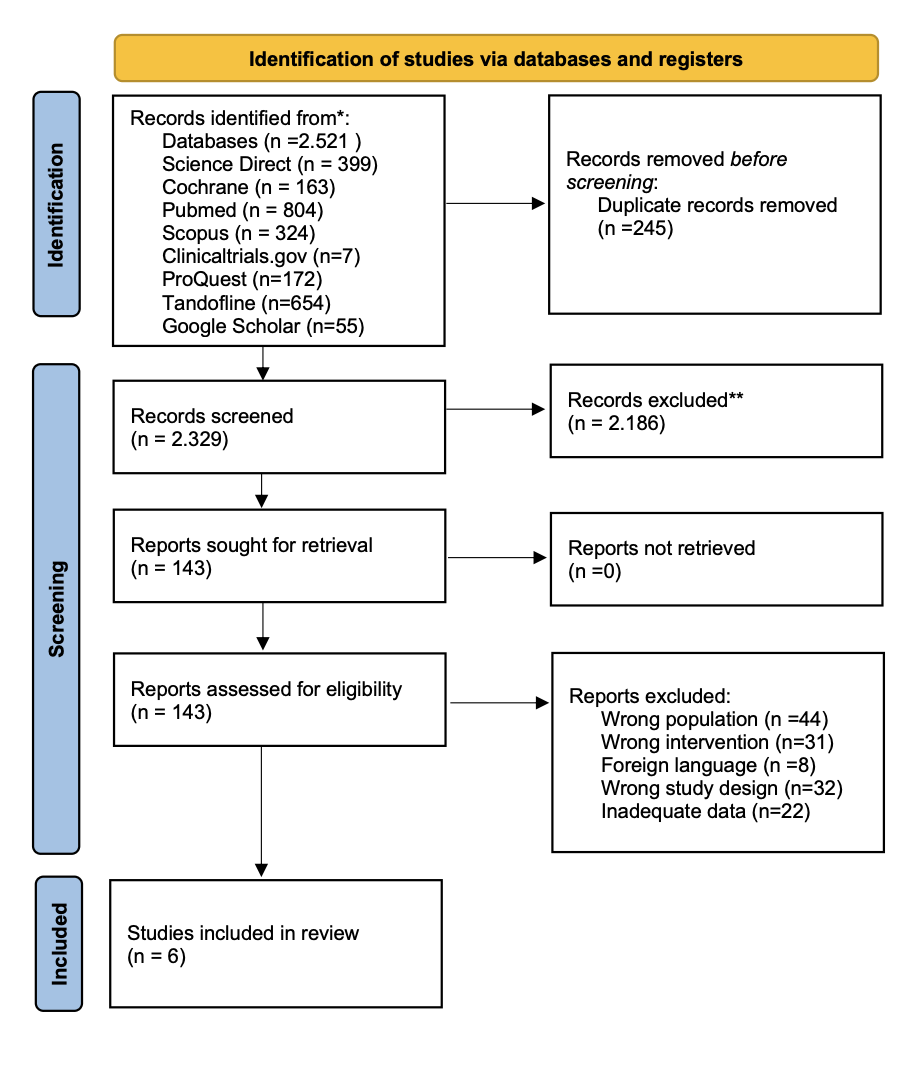


Figure 1. PRISMA diagram of included studies in the systematic review

| **A. Case Reports** | | | |
| --- | --- | --- | --- |
| **JBI Item** | **Wang 2020** | **Cascella 2021** | **Corripio 2016** |
| 1. Demographics | Yes | Yes | Yes |
| 2. History & timeline | Yes | Yes | Yes |
| 3. Clinical condition | Yes | Yes | Yes |
| 4. Diagnostic tests |  |  |  |
| 5. Intervention |  |  |  |
| 6. Post-intervention condition |  |  |  |
| 7. Adverse events |  |  |  |
| 8. Takeaway lessons | Yes | Yes | Yes |
| **B. Case Series** | | | |
| **JBI Item** | **Aibar-Durán 2023** | **Bioque 2025** | **Roldán 2020** |
| 1. Clear inclusion criteria |  |  |  |
| 2. Standard measurement |  |  |  |
| 3. Valid identification |  |  |  |
| 4. Consecutive inclusion |  |  |  |
| 5. Complete inclusion |  |  |  |
| 6. Demographics |  |  |  |
| 7. Clinical info |  |  |  |
| 8. Valid outcomes |  |  |  |
| 9. Site/clinicians clear |  |  |  |
| 10. Statistics appropriate |  |  |  |
| Green=Available; Yellow=Unclear; Red=Unavailable | | | |

Table 2. Risk of Bias using JBI Scoring

| No | Author | PANSS Baseline | | | | PANSS Post DBS | | | | PANSS Improvement | | | | |  | CGI | | BPRS | |
| --- | --- | --- | --- | --- | --- | --- | --- | --- | --- | --- | --- | --- | --- | --- | --- | --- | --- | --- | --- |
|  |  | P | N | G | T | P | N | G | T | P | N | G | T | % | 100 | Baseline | Post-DBS | Base-line | Post-DBS |
| 1 | Corripio (2016) | 13 | 18 | NA | NA | 5 | 12 | NA | NA | -8 | -6 | NA | NA | NA |  | NA | NA | NA | NA |
| 2a | Bioque (2025) | NA | NA | NA | 140 | NA | NA | NA | 100 | NA | NA | NA | -40 | -28,6 |  | 6 | 4 | NA | NA |
| 2b |  | NA | NA | NA | 91 | NA | NA | NA | 79 | NA | NA | NA | -12 | -13,2 |  | 5 | 3 | NA | NA |
| 2c |  | NA | NA | NA | 107 | NA | NA | NA | 91 | NA | NA | NA | -16 | -14,9 |  | 5 | 4 | NA | NA |
| 3a | Aibar-Durán (2023) | 27 | 24 | 46 | 97 | 20 | 24 | 41 | 86 | -7 | 0 | -5 | -11 | -11,3 |  | NA | NA | NA | NA |
| 3b |  | 24 | 29 | 49 | 102 | 18 | 26 | 33 | 78 | -6 | -3 | -16 | -24 | -23,5 |  | NA | NA | NA | NA |
| 3c |  | 17 | 12 | 36 | 65 | 16 | 10 | 26 | 53 | -1 | -2 | -10 | -12 | -18,5 |  | NA | NA | NA | NA |
| 3d |  | 21 | 19 | 44 | 84 | 15 | 15 | 39 | 70 | -6 | -4 | -5 | -14 | -16,7 |  | NA | NA | NA | NA |
| 4a | Roldán (2020) | 27 | 24 | 46 | 97 | 9 | 18 | 23 | 50 | -18 | -6 | -23 | -47 | -48,5 |  | NA | NA | NA | NA |
| 4b |  | 24 | 29 | 49 | 102 | 14 | 26 | 31 | 71 | -10 | -3 | -18 | -31 | -30,4 |  | NA | NA | NA | NA |
| 4c |  | 17 | 12 | 36 | 65 | 7 | 8 | 20 | 35 | -10 | -4 | -16 | -30 | -46,2 |  | NA | NA | NA | NA |
| 4d |  | 26 | 28 | 54 | 108 | 17 | 18 | 44 | 79 | -9 | -10 | -10 | -29 | -26,8 |  | NA | NA | NA | NA |
| 4e |  | 22 | 23 | 39 | 84 | 16 | 13 | 18 | 47 | -6 | -10 | -21 | -37 | -44,1 |  | NA | NA | NA | NA |
| 4f |  | 27 | 21 | 37 | 85 | 21 | 30 | 35 | 86 | -6 | 9 | -2 | 1 | 1,2 |  | NA | NA | NA | NA |
| 5a | Aibar-Durán (2023) | 26 | 28 | 54 | 108 | 20 | 25 | 41 | 86 | -6 | -3 | -13 | -22 | -20,4 |  | NA | NA | NA | NA |
| 5b |  | 19 | 20 | 34 | 73 | 20 | 25 | 34 | 78 | 1 | 5 | 0 | 5 | 6,8 |  | NA | NA | NA | NA |
| 5c |  | 22 | 32 | 33 | 87 | 15 | 22 | 29 | 67 | -7 | -10 | -4 | -20 | -22,9 |  | NA | NA | NA | NA |
| 5d |  | 22 | 23 | 39 | 84 | 16 | 19 | 27 | 61 | -6 | -4 | -12 | -23 | -27,4 |  | NA | NA | NA | NA |
| 6 | Cascella (2021) | NA | NA | NA | NA | NA | NA | NA | NA | NA | NA | NA | NA | NA |  | NA | NA | 42 | 20 |
| 7a | Wang (2020) | 13 | 23 | 38 | 74 | 22 | 24 | 35 | 81 | 9 | 1 | -3 | 7 | 9,5 |  | NA | NA | NA | NA |
| 7b |  | 26 | 41 | 53 | 120 | 12 | 28 | 42 | 82 | -14 | -13 | -11 | -38 | -31,7 |  | NA | NA | NA | NA |
|  | Mean | 21.94 | 23,88 | 42.94 | 93.32 | 15.47 | 20.18 | 32.38 | 72.63 | -6.47 | -3.71 | -10.56 | -20.68 | -21,5 |  | 5.33 | 3.67 | NA | NA |
| P= Positive; N=Negative; G=General; T=Total;NA=Not Available;PANSS=Positive and Negative Syndrome Scale;CGI=Clinical Global Impression;BPRS=Brief Psychiatric Rating Scale | | | | | | | | | | | | | | | | | | | |

Table 3. PANSS Score of each patients

| **Author (Year)** | **Brain Target** | **Targeting Method & Imaging** | **Stereotactic Coordinates (x,y,z in mm)** | **Lead Model & Hardware** | **Intraoperative Confirmation** | **Surgical Complications** |
| --- | --- | --- | --- | --- | --- | --- |
| Corripio et al. (2016) | NAcc | MRI-Direct Visual | x:±7,y:6,z:−4 (relative to AC) | Not specified | Macro-stimulation (test for akathisia) | None reported |
| Bioque et al. (2025) | NAcc | 3T MRI-Direct; StealthStation S8 | Not explicitly listed in text | Medtronic 3389-40 (Tetra-polar) | MRI-guided & intraop image verification | No surgery-related complications |
| Aibar-Durán et al. (2023) | NAcc & SCG | MRI-CT Merged; Lead-DBS Toolbox | NAcc: 7.3,6.4,−4.5; SCG: 5,26,−11 | Medtronic 3387S-28/40; Activa PC IPG | Merged image verification (Lead-DBS) | 1 Infection (required system removal) |
| Roldán et al. (2020) | NAcc & sgACC | T1-MRI structural; SPM12 for PET | Not listed (Coordinates in native space) | Not specified | Clinical assessment (6-month post-op) | 1 Hemorrhage + Infection (lead removal) |
| Cascella et al. (2021) | SNr | ROSA Robotic Guidance | x:±12.5,y:−4,z:−13 (relative to MCP) | Medtronic 3387 | Stereotactic frame verification | Significant weight gain (33 lbs) |
| Wang et al. (2020) | Habenula (HB) | 3.0T MRI; QSM imaging | x:4.5,y:−25.5,z:1.5 (relative to MCP) | Medtronic 3389 / PINS L302 | Acute stimulation testing (numbness/pain) | No surgical complications |

Table 4. Surgical aspect and technique in each included study

| Case | Author, Year | Patient Demographics | DBS Target | Stimulation Parameters | Follow-up Duration | Secondary Outcomes |
| --- | --- | --- | --- | --- | --- | --- |
| 1 | Corripio et al. (2016) | Female, 46 years old, 24-year illness duration, treatment-resistant schizophrenia | Bilateral nucleus accumbens (NAcc) | Unilateral stimulation: 2.5-3.5V, 130Hz, 60μs pulse width | 11 months | Disorganized symptoms: 50% reduction; Excited symptoms: 66.7% reduction |
| 2 | Miquel Bioque et al. (2025) | Male, 52 years old,illness duration 32 years | Bilateral nucleus accumbens (NAcc) | 130Hz, 90μs pulse width, 0.5-7.5V bipolar stimulation | 12 months | Discontinued maintenance ECT in 3/4 original patients; Improved depression scores; Digital monitoring data |
| 3 | Miquel Bioque et al. (2025) | Female, 48 years old, illness duration 27 years |  |  |  |  |
| 4 | Miquel Bioque et al. (2025) | Male, 46 years old,illness duration 19 years |  |  |  |  |
| 5 | Aibar-Durán et al. (2023) | 5 females, 3 males; median age 43 years; 7 ultra-treatment-resistant patients | Bilateral nucleus accumbens (NAcc) | 3.5-7mA amplitude, 60-210μs pulse width, 120-210Hz frequency | 36 months | GAF scale changes; Connectomic analysis; Volume of Activated Tissue (VAT) modeling |
| 6 | Aibar-Durán et al. (2023) |  |  |  |  |  |
| 7 | Aibar-Durán et al. (2023) |  |  |  |  |  |
| 8 | Aibar-Durán et al. (2023) |  |  |  |  |  |
| 9 | Roldán et al. (2020) | Female, 46 years old, illness duration 24 years | Bilateral nucleus accumbens (NAcc) | 2.5-7.5V amplitude, 60-210μs pulse width, 120-210Hz frequency |  | Brain glucose metabolism changes measured with PET imaging |
| 10 | Roldán et al. (2020) | Male, 43 years old, illness duration 11 years |  |  |  |  |
| 11 | Roldán et al. (2020) | Female, 35 years old, illness duration 10 years |  |  |  |  |
| 12 | Roldán et al. (2020) | Male, 34 years old, illness duration 9 years | sgACC (subgenual anterior cingulate cortex) |  |  |  |
| 13 | Roldán et al. (2020) | Female, 53 years old, illness duration 23 years |  |  |  |  |
| 14 | Roldán et al. (2020) | Female, 38 years old, illness duration 21 years |  |  |  |  |
| 15 | Aibar-Durán et al. (2023) | 5 females, 3 males; median age 43 years; 7 ultra-treatment-resistant patients | SCG (subgenual cingulate gyrus) | 3.5-7mA amplitude, 60-210μs pulse width, 120-210Hz frequency |  | GAF scale changes; Connectomic analysis; Volume of Activated Tissue (VAT) modeling |
| 16 | Aibar-Durán et al. (2023) |  |  |  |  |  |
| 17 | Aibar-Durán et al. (2023) |  |  |  |  |  |
| 18 | Aibar-Durán et al. (2023) |  |  |  |  |  |
| 19 | Cascella et al. (2021) | Female, 35 years old, treatment-resistant paranoid schizophrenia | Bilateral Substantia nigra pars reticulata (SNr) | Left: 1.0V, Right: 0.8V, 130Hz, 60μs pulse width | 6 months (1-year follow-up) | BPRS Total: Clinically significant improvement; Halucinatoins & unusual thought content: clinically significant. Improved negative symptoms (avolition, anhedonia); Enhanced verbal fluency; Declined memory performance |
| 20 | Wang et al. (2020) | Male, 26 years old, illness duration 9 years | Bilateral habenula (HB) | Case 1: 2.0V, 60μsec, 60Hz (left); 2.5V, 60μsec, 60Hz (right) | 10 months | Acute stimulation effects documented in both patients |
| 21 | Wang et al. (2020) | Male, 21 years old, illness duration 4 years |  | Case 2: 3.15V, 80μsec, 135Hz (left); 3.2V, 60μsec, 135Hz (right) | 12 months |  |

Table 5. Secondary outcomes of included studies

| **DBS Target** | **Studies** | **Total Patients** | **Response Rate** | **Key Clinical Benefits** | **Limitations** | **Source** |
| --- | --- | --- | --- | --- | --- | --- |
| **Nucleus Accumbens (NAcc)** | 4 studies | 11 patients | 100% (11/11) | Broad symptom improvement, ECT discontinuation possible | Variable negative symptom response | Corripio et al. (2016), Miquel Bioque et al. (2025), Aibar-Durán et al.(2023), Roldán et al. (2020) |
| **Substantia Nigra (SNr)** | 1 study | 1 patient | 100% (1/1) | • Complete psychosis resolution, Immediate effect,Low voltage requirements | Single case | Cascella et al. (2021) |
| **Habenula (HB)** | 1 study | 2 patients | 100% (2/2) | Neurophysiological validation, Reward/aversion processing | Mixed clinical outcomes,Limited durability | Wang et al. (2020) |
| **Subgenual Cingulate (SCG)** | 1 study | 4 patients | 75% (3/4) | Moderate symptom reduction, Different connectivity patterns | • Lower response compared to NAcc Limited efficacy data | Aibar-Durán et al.(2023) |
| **sgACC (subgenual anterior cingulate cortex)** | 1 study | 3 patients | 100% (3/3) | Psychotic symptoms reduction | sgACC-DBS reduced, while NAc-DBS increased, metabolic activity in the NAc, hippocampus, thalamus, and prefrontal cortex. 1 patient didn’t show improvement | Roldán et al. (2020) |

Table 6. Target-Specific Clinical Outcome

| **Safety Parameter** | **Incidence** | **Severity** | **Management** | **Clinical Impact** | **Source** |
| --- | --- | --- | --- | --- | --- |
| **Surgical Complications** | 8.7% (2/23) | Moderate to severe | Electrode removal | Acceptable for treatment-resistant population | Aibar-Durán et al. (2023), Roldán et al.(2020) |
| **Infection** | 4.3% (1/23) | Severe | Antibiotic treatment + removal | Standard neurosurgical risk | Roldán et al. (2020) |
| **Hemorrhage** | 4.3% (1/23) | Severe | Neurosurgical intervention | Within expected DBS risk range | Roldán et al. (2020) |
| **Stimulation Side Effects** | 8.7% (2/23) | Mild to moderate | Parameter adjustment | Generally manageable | Wang et al. (2020) |
| **Weight Gain** | 4.3% (1/23) | Moderate | Dietary management | Monitor in NAcc/reward targets | Cascella et al. (2021) |

Table 7. Safety and Tolerability Profile


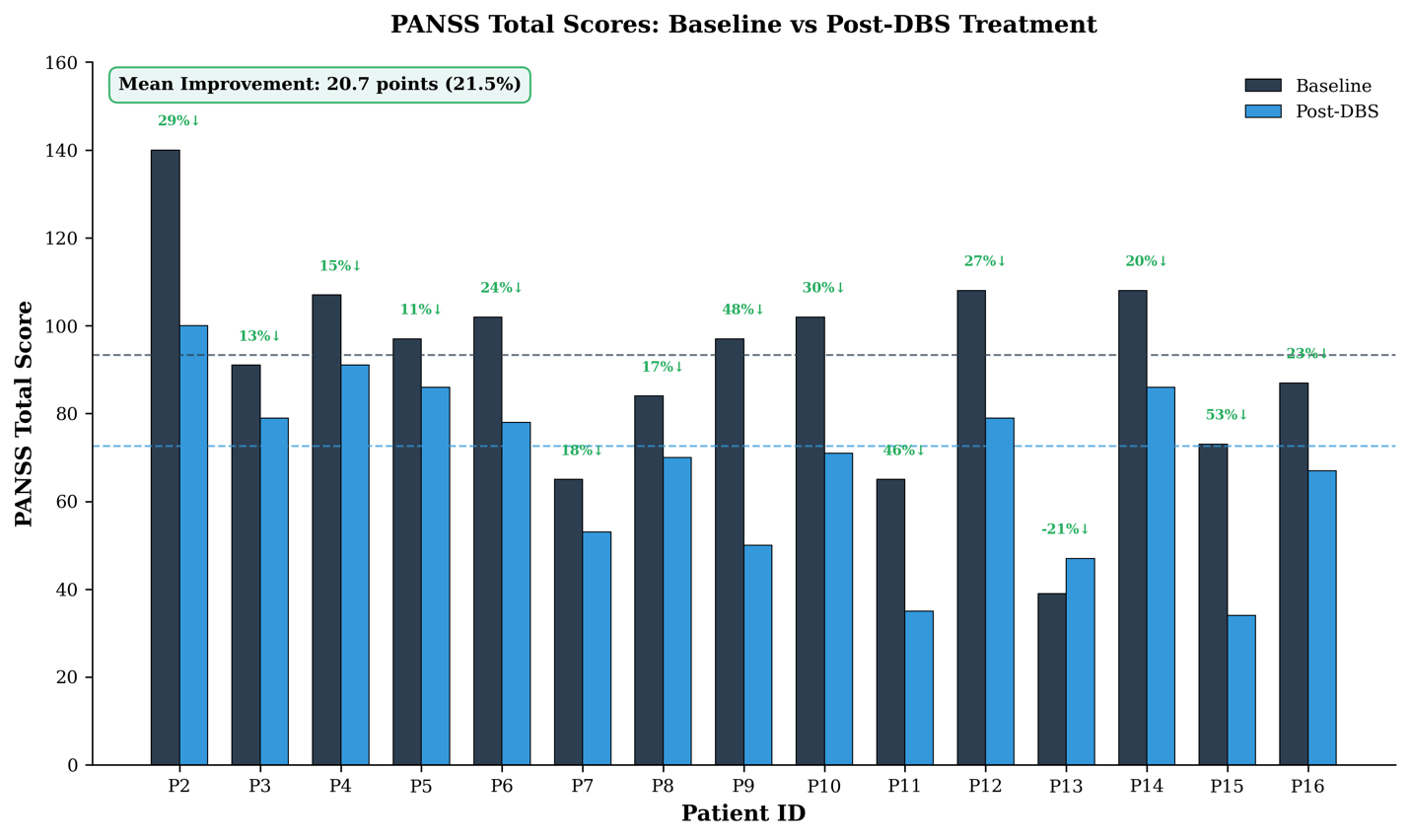


Figure 2. Baseline PANSS and Post-DBS treatment
